# The sequence of regional structural disconnectivity due to multiple sclerosis lesions

**DOI:** 10.1093/braincomms/fcad332

**Published:** 2023-12-05

**Authors:** Ceren Tozlu, Emily Olafson, Keith W Jamison, Emily Demmon, Ulrike Kaunzner, Melanie Marcille, Nicole Zinger, Nara Michaelson, Neha Safi, Thanh Nguyen, Susan Gauthier, Amy Kuceyeski

**Affiliations:** Department of Radiology, Weill Cornell Medicine, NewYork, NY, 10065, USA; Department of Radiology, Weill Cornell Medicine, NewYork, NY, 10065, USA; Department of Radiology, Weill Cornell Medicine, NewYork, NY, 10065, USA; Department of Neurology, Weill Cornell Medical College, NewYork, NY, 10065, USA; Department of Neurology, Weill Cornell Medical College, NewYork, NY, 10065, USA; Department of Neurology, Weill Cornell Medical College, NewYork, NY, 10065, USA; Department of Neurology, Weill Cornell Medical College, NewYork, NY, 10065, USA; Department of Neurology, Weill Cornell Medical College, NewYork, NY, 10065, USA; Department of Neurology, Weill Cornell Medical College, NewYork, NY, 10065, USA; Department of Radiology, Weill Cornell Medicine, NewYork, NY, 10065, USA; Department of Radiology, Weill Cornell Medicine, NewYork, NY, 10065, USA; Department of Neurology, Weill Cornell Medical College, NewYork, NY, 10065, USA; Department of Radiology, Weill Cornell Medicine, NewYork, NY, 10065, USA

**Keywords:** multiple sclerosis, disease progression, cognition, chronic active lesions, event-based modelling

## Abstract

Prediction of disease progression is challenging in multiple sclerosis as the sequence of lesion development and retention of inflammation within a subset of chronic lesions is heterogeneous among patients. We investigated the sequence of lesion-related regional structural disconnectivity across the spectrum of disability and cognitive impairment in multiple sclerosis. In a full cohort of 482 multiple sclerosis patients (age: 41.83 ± 11.63 years, 71.57% females), the Expanded Disability Status Scale was used to classify patients into (i) no or mild (Expanded Disability Status Scale <3) versus (ii) moderate or severe disability groups (Expanded Disability Status Scale ≥3). In 363 out of 482 patients, quantitative susceptibility mapping was used to identify paramagnetic rim lesions, which are maintained by a rim of iron-laden innate immune cells. In 171 out of 482 patients, Brief International Cognitive Assessment was used to identify subjects as being cognitively preserved or impaired. Network Modification Tool was used to estimate the regional structural disconnectivity due to multiple sclerosis lesions. Discriminative event-based modelling was applied to investigate the sequence of regional structural disconnectivity due to (i) all representative T2 fluid-attenuated inversion recovery lesions, (ii) paramagnetic rim lesions versus non-paramagnetic rim lesions separately across disability groups (‘no to mild disability’ to ‘moderate to severe disability’), (iii) all representative T2 fluid-attenuated inversion recovery lesions and (iv) paramagnetic rim lesions versus non-paramagnetic rim lesions separately across cognitive status (‘cognitively preserved’ to ‘cognitively impaired’). In the full cohort, structural disconnection in the ventral attention and subcortical networks, particularly in the supramarginal and putamen regions, was an early biomarker of moderate or severe disability. The earliest biomarkers of disability progression were structural disconnections due to paramagnetic rim lesions in the motor-related regions. Subcortical structural disconnection, particularly in the ventral diencephalon and thalamus regions, was an early biomarker of cognitive impairment. Our data-driven model revealed that the structural disconnection in the subcortical regions, particularly in the thalamus, is an early biomarker for both disability and cognitive impairment in multiple sclerosis. Paramagnetic rim lesions–related structural disconnection in the motor cortex may identify the patients at risk for moderate or severe disability in multiple sclerosis. Such information might be used to identify people with multiple sclerosis who have an increased risk of disability progression or cognitive decline in order to provide personalized treatment plans.

## Teaser

Tozlu *et al*. report that subcortical structural disconnectivity due to lesions identified with T2 fluid-attenuated inversion recovery occurs earlier across the spectrum of disability and cognitive impairment in people with multiple sclerosis. Paramagnetic rim lesion (PRL)–based structural disconnectivity in motor-related regions occurs earlier compared to non–PRL-based structural disconnectivity in the disability event sequence.

## Graphical Abstract

**Graphical Abstract:**
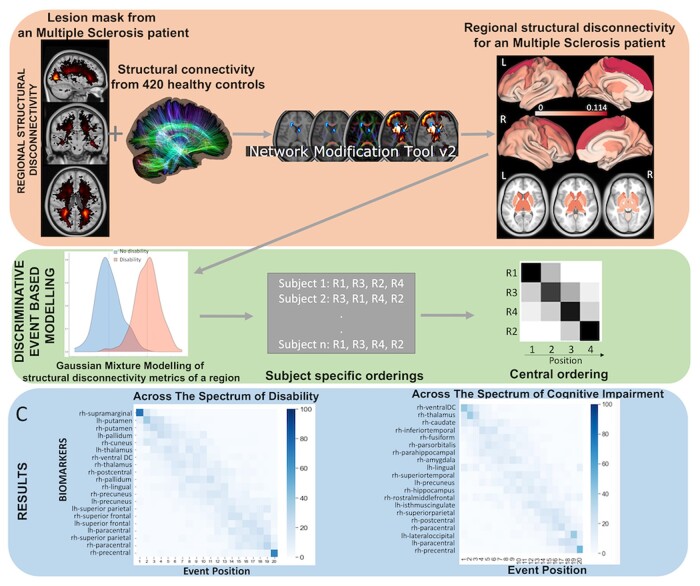

## Introduction

The size, location and underlying pathology of white matter (WM) lesions are heterogeneous among people with multiple sclerosis,^1^ making the prediction of disease progression challenging. Moreover, the sequence in which lesions disrupt various brain regions’ structural connectivity (WM streamlines) and how these disruptions impact disability progression and/or cognitive impairment in multiple sclerosis have not yet been studied. Discriminative event-based modelling (dEBM) is an approach used to estimate the temporal sequence of biomarker abnormalities over disease progression using cross-sectional data.^2-7^ While the exact timing of an event (i.e. a biomarker becomes abnormal) or the time between the events cannot be identified due to non-linear appearance of the events and utilization of a cross-sectional data, dEBM provides the relative order of the events. This data-driven approach has been used to investigate the sequence of grey matter (GM) atrophy only^2^ or to investigate the sequence of changes in a combination of features like atrophy, lesion load, properties of the brain’s functional networks and microstructure of major WM tracts^3^ across the stages of motor and cognitive impairment in multiple sclerosis. However, these studies restricted the structural biomarkers by using *a priori*–defined regions of interest; here, we begin with whole-brain biomarkers selected in a data-driven manner.

Motor and cognitive impairments in multiple sclerosis have been related to disruptions in the network of WM tracts in the brain, referred to collectively as the brain’s structural disconnectivity.^4,5^ The Network Modification (NeMo) Tool,^6^ which measures structural disconnectivity due to lesions, has been used by our group and others to correlate lesion-related structural disconnectivity patterns to impairments, outcomes, functional connectivity disruptions, rehabilitation response and GM pathology in people with (pw) multiple sclerosis.^4,7-10^ Importantly, our recent study of pw multiple sclerosis demonstrated the clinical utility of the NeMo Tool in understanding mechanisms of disease by showing that the structural disconnectivity estimated using the NeMo Tool was more predictive of disability than structural connectivity measured using an individual’s diffusion MRI (dMRI).^8^

In addition to their size and location, a lesion’s underlying pathology can also influence its impact on disability. While fluid-attenuated inversion recovery (FLAIR) images provide valuable insights about the overall burden of the disease and neurobiological changes such as demyelination, axonal loss and inflammation, quantitative susceptibility mapping (QSM) can provide complementary information and improve our understanding on the neurodegeneration and chronic central nervous system (CNS) inflammation by measuring iron deposition and microglial activation, which exist in chronic active lesions. Chronic CNS inflammation in the multiple sclerosis lesion is maintained, in part, with iron-laden pro-inflammatory microglia/macrophages at the rim of chronic active multiple sclerosis lesions.^11-13^ QSM^14,15^ has been used to identify paramagnetic rim lesions (PRLs)^16^ and has expanded our understanding of chronic active lesions on disease mechanisms and disease progression.^16-19^ Structural disconnectivity due to PRL has been shown to be better associated with disability as compared to structural disconnectivity due to non-PRL.^5^ However, it remains unknown if the sequence of regional structural disconnectivity differs among PRL compared to non-PRL and if this difference impacts clinical disability.

Here, we use dEBM to investigate the sequence of regional structural disconnectivity due to all multiple sclerosis lesions (as identified on T2 FLAIR), PRL and non-PRL, estimated using the NeMo Tool, as disability and cognitive impairment range from less to more severe. Understanding the impact of the lesion size, location and classification on multiple clinical outcomes may provide an opportunity to identify the patients at risk for future disability and allow personalized treatments to minimize the burden of multiple sclerosis.

## Materials and methods

### Subjects

Four hundred eighty-two pw multiple sclerosis (age: 41.83 ± 11.63 years, 71.57% females; the ‘±’ sign indicates standard deviation) with a diagnosis of clinically isolated syndrome (CIS) (*n* = 16) or multiple sclerosis (430 relapsing–remitting and 36 primary or secondary progressive multiple sclerosis) were enrolled in our retrospective study; inclusion criteria included no contraindications to MRI. Demographic data were collected (age, sex, clinical phenotype and disease duration), and subjects underwent MRI. Extended Disability Status Score (EDSS) was used to quantify disability, where an EDSS < 3 was considered no and mild disability and EDSS ≥3 was considered moderate to severe disability.^20^ All studies were approved by an ethical standards committee of Weill Cornell Medicine on human experimentation, and written informed consent was obtained from all patients.

The Brief International Cognitive Assessment for Multiple Sclerosis (BICAMS) was used to assess cognition in a subset of 171 pw multiple sclerosis. These 171 patients were already participants within our research repository that were willing (and reconsented) to have an annual cognitive evaluation at the time of their annual MRI for a total of 5 years. BICAMS consists of three assessments: (i) the Symbol Digit Modalities Test (SDMT), which measures the processing speed; (ii) California Verbal Learning Test-II (CVLT-II) immediate recall (total of Trials 1–5), which measures verbal learning and short-term memory; and (iii) Brief Visuospatial Memory Test-Revised (BVMT-R) immediate recall (total of Trials 1–3), which measures visuospatial learning and short-term memory.^21^ A higher score of each test is associated with better cognitive performance. If a subject’s SDMT *Z*-score was < −1 (21% of 171 pw multiple sclerosis) or CVLT or BVMT *t*-scores were <40 (10% of 171 pw multiple sclerosis for CVLT and 23% of 171 pw multiple sclerosis for BVMT), they were considered significantly impaired on that test.^21^ In this paper, we identified pw multiple sclerosis to be cognitively impaired (CI) if they showed impairments in at least two cognitive tests; otherwise, they were considered cognitively preserved (CP).^22^

### Ethics statement

All studies were approved by an ethical standards committee of Weill Cornell Medicine on human experimentation, and written informed consent was obtained from all patients.

### Image acquisition, processing and connectome extraction

MRI images were obtained on two different scanners: Siemens and GE. The Siemens and GE scanning protocol was performed accordingly to the protocol performed by Zinger *et al.*^23^ Two hundred seventy-nine patients were scanned on Siemens and 203 patients were scanned on GE. Both GE and Siemens scanners were 3 T. The T2 FLAIR and QSM images were used only to extract the lesion masks and identify the PRLs. The values from these images, which may differ based on the scanner, were not used in this study. Additionally, both scanners had similar parameters for the axial 3D multi-echo gradient recalled echo (GRE) sequence for QSM: axial field of view = 24 cm, repetition time/echo time (TE)1/ΔTE = 48.0/6.3/4.1 ms, number of TEs = 10, fractional anisotropy = 15°, GRAPPA parallel imaging factor (*R*) = 2, voxel size = 0.75 × 0.93 × 3 mm^3^, and scan time = 4.2 min using a fully automated morphology enabled dipole inversion (MEDI+0) method zero referenced to the ventricular CSF.^24^ The QSM protocol has been harmonized for both scanner manufacturers and was demonstrated to be reproducible across manufacturers.^15^

### Lesion mask creation

The conventional images (T1w, T2w and T2w FLAIR) were co-registered to the sum-of-squares echo-combined magnitude GRE images using the FMRIB’s Linear Image Registration Tool algorithm^25^; automated brain segmentation was performed using FreeSurfer.^26^ WM tissue segmentations were manually edited for misclassification due to WM T_1_ hypointensities associated with lesions. The WM hyperintensity lesion masks were created from the T2 FLAIR images by categorizing the tissue type based on the image intensities within the Lesion Segmentation Tool using the lesion prediction algorithm (LPA) method; the masks generated were further hand edited if necessary. Next, the T2 FLAIR lesions masks were co-registered to the QSM images and further hand edited (if needed) to better match the lesion geometry on QSM. A lesion was designated as PRL if QSM was hypointense core and hyperintense at the edge of the lesion in either a complete or partial manner. The presence of a partial and/or full hyperintense QSM rim (i.e. PRL) was determined as a consensus of two trained reviewers, and in the case of disagreement, an independent third reviewer decided on the presence of a positive hyperintense rim. Once the PRLs were identified, they were removed from the T2 FLAIR lesion masks to obtain a non-PRL mask.

Lesion masks were transformed to the individual’s T_1_ native space using the inverse of the T_1_ to GRE transform and nearest neighbour interpolation. Individual T_1_ images were then normalized to 1 mm MNI152 v6 space using FSL’s linear (FLIRT) and non-linear (FNIRT) transformation tools (http://www.fmrib.ox.ac.uk/fsl/index.html); transformations with nearest neighbour interpolation were then applied to transform the native anatomical space T2 FLAIR lesion masks to MNI space. The transformations were concatenated (T2 FLAIR to T_1_ to MNI) to minimize interpolation effects. Lesions were visually inspected after the transformation to MNI space to verify the accuracy of co-registration. QSM was available in 363 pw multiple sclerosis to allow for designation of individual chronic lesions as a PRL or non-PRL. Forty-three per cent (156/363) of these patients had ≥1 PRL; therefore, separate PRL and non-PRL (T2 FLAIR lesions minus PRL) lesion masks were created for these subjects.

### NeMo Tool

The MNI space T2 FLAIR lesions (for all subjects), PRL (for the subjects who had ≥1 PRL) and non-PRL (for the subjects who had ≥1 PRL) masks were processed through the newest version of the NeMo Tool,^6^ NeMo Tool 2.0, that estimates a lesion mask’s subsequent pattern of structural disconnectivity. The pattern of structural disconnectivity metric was defined for 68 cortical (from the Desikan–Killany atlas), and 18 subcortical and cerebellar regions as the per cent of tractography streamlines connecting that region that also pass through the lesion mask. The newest version of the tractography database consists of structural connectomes from 420 unrelated healthy controls (206 females, 214 males, 28.7 ± 3.7 years^8^; see Supplementary material for details). Structural disconnectivity metrics were calculated separately using PRL and non-PRL masks as well as for the complete T2 FLAIR lesion masks. The structural disconnectivity metrics from the T2 FLAIR lesion mask were computed across all subjects (*N* = 482), while the structural disconnectivity metric scores from PRL versus non-PRL masks were computed only for the subjects that had QSM imaging (*N* = 363). Those subjects having QSM imaging but no PRL were assigned PRL structural disconnectivity scores of 0, since there was no structural disconnectivity due to PRL for these subjects.

### dEBM

**Figure 1:**
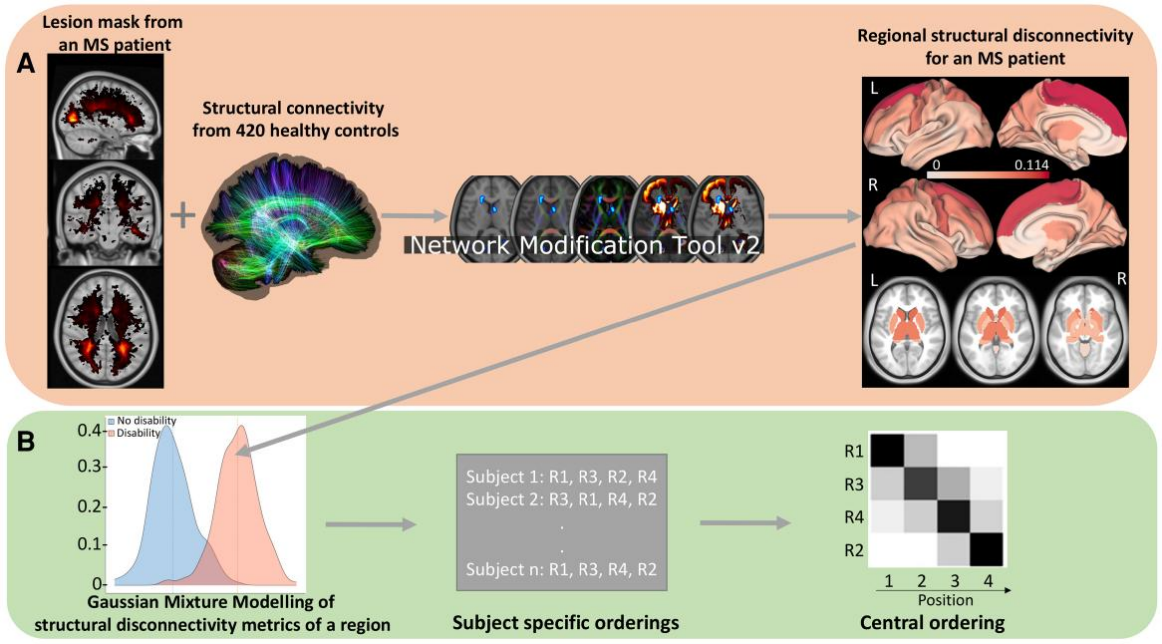
**Workflow.** (**A**) The NeMo tool was used to predict regional structural disconnectivity metrics from the pw multiple sclerosis lesion masks. (**B**) The dEBM algorithm was applied to the regional structural disconnectivity metrics to estimate the sequence of regional structural disconnections as disability/cognitive impairment occurs. First, a GMM was used to calculate the probability distributions of the regional structural disconnectivity metrics for two groups (for example no disability versus disability groups). Second, the likelihood defined in the text is maximized by finding the optimal sequence of regional structural disconnection ‘events’. Results are visualized via a matrix with rows indicating regional structural disconnectivity and columns indicating the position in the sequence of disability/cognitive impairment progression. The intensity of each matrix entry corresponds to the certainty of that region’s structural disconnection at that position in the sequence across 100 bootstraps.

We used dEBM applied to cross-sectional data to estimate the most likely sequence in which regions appear increasingly disconnected across the spectrum from no/mild disability to moderate/severe disability or from CP to CI (see Fig. 1).^27,28^ The conversion of a metric (in this case regional structural disconnection) from healthy to pathological is called as event ($E_{pn}$), where *n* indicates the subject n=1,2,…,N and *p* indicates the region p=1,2,…,P. First, dEBM estimates the posterior probability of each region being within healthy range (i.e. less disconnected) versus pathological (i.e. more disconnected) using a Gaussian mixture model (GMM) of the two subject groups’ distributions of regional structural disconnectivities. The GMM allows calculation of the likelihood that region *p* of subject *n* is pathological, i.e. $P(x_{pn}|E_{n})$ (i.e. the likelihood that the structural disconnection has occurred on region *p* for subject *n*), or healthy $P(x_{pn}|-E_{n})$ (i.e. the likelihood that the structural disconnection has not occurred in region *p* for subject *n*). Second, the sequence (*S*) of the events (i.e. regions becoming pathologically disconnected) is created by maximizing the following likelihood:


$$P(X|S)=\overset{N}{\underset{n=1}{\prod }}\;\left[\overset{P}{\underset{k=0}{\sum }}\;(P(k)\overset{k}{\underset{p=1}{\prod }}\;P(x_{pn}|E_{n})\overset{P}{\underset{p=k+1}{\prod }}\;P(x_{pn}|-E_{n}))\right],$$

where *X* is the data matrix containing the regional disconnectivity scores for all subjects and *P*(*k*) is the prior probability of being at stage *k*, which indicates that events $E_{1},\;\ldots ,\;E_{k}$ have occurred but $E_{k+1},\;\ldots ,\;E_{P}$ have not yet occurred. The entire algorithm is repeated using 100 bootstrapped samples of the data to compute the uncertainty (i.e. the positional variance of the sequence) and standard error of the event centres. dEBM results are visualized via a matrix with rows indicating regions and columns indicating position in the sequence of disability/cognitive impairment progression. The intensity of each matrix entry corresponds to the certainty of that region’s structural disconnection at that position in the sequence across 100 bootstraps (darker colour with greater certainty). The codes to perform the dEBM analysis are publicly available (https://github.com/EuroPOND/pyebm).

In our study, we applied the dEBM algorithm to three different data sets (see Supplementary Fig. 1 for the flow diagram):

1. Regional structural disconnectivity due to T2 FLAIR lesions in all subjects (*N* = 482) across the spectrum from no or mild disability (EDSS < 3; *N* = 379) to moderate or severe disability (EDSS ≥3; *N* = 103)
2. Regional structural disconnectivity due to PRL versus non-PRL separately in a subset of the subjects (*N* = 363) across the spectrum from no or mild disability (EDSS < 3; *N* = 272) to moderate or severe disability (EDSS ≥3; *N* = 91)
3. Regional structural disconnectivity due to T2 FLAIR lesions in a subset of the subjects (*N* = 171) across the spectrum from CP (*N* = 150) to CI (*N* = 21)
4. Regional structural disconnectivity due to PRL versus non-PRL separately in a subset of the subjects (*N* = 171) across the spectrum from CP (*N* = 150) to CI (*N* = 21)

For each data set, we compared the regional structural disconnectivity metrics between disability or cognition groups (each domain was calculated separately) using Student’s *t*-test. First, we repeated Student’s *t*-test using 100 bootstrap samples of the original data set. Second, the rank of each region was calculated for each bootstrap sample. Third, we computed the average of the ranks over 100 bootstrap iterations for each region. Finally, we used the top 20 regions that showed the highest averaged rank across 100 bootstrap samples in the dEBM algorithms to reduce the dimensionality of the model. To control for confounding variables, covariates age and sex were regressed out of each structural disconnectivity metric using a linear regression model before inclusion in the dEBM.

To investigate if the sequence of the regional structural disconnectivity metrics is different from random, where each region has 1/20 probability of being in each order, we compared the per cent of times a region was assigned its final order across the 100 bootstraps with 1/20 using one-sample Student’s *t*-test.

### Statistical analysis

First, demographics and clinical variables were tested for differences between the no/mild disability versus moderate/severe disability groups and the CP versus CI groups using the *χ*^2^ test for qualitative variables and Wilcoxon rank-sum test for quantitative variables. The normality of the structural disconnectivity metrics was checked using Shapiro–Wilk test. We found that the structural disconnectivity was following a non-normal distribution for all regions with an average of the *P*-values 6.680e−18 and *W*-values 0.66 from Shapiro–Wilk test. Therefore, Student’s *t*-tests were used to quantify group differences in the regional structural disconnectivity metrics (after logarithmic transformation to ensure normality). Differences were considered significant when *P* < 0.05 after Benjamini–Hochberg (BH) correction for multiple comparisons.^29^ All statistical analyses were performed, and graphs were created using R version 3.4.4, MATLAB version R2021b and Python version 3.9.

## Results

### Patient characteristics

Table 1 shows the demographics, clinical and imaging features of all subjects and by disability (no/mild versus moderate/severe disability), having PRL (at least one PRL versus no PRL) and cognition (CP versus CI) groups. The pw multiple sclerosis who had moderate to severe disability were older (*t* = 6.31, *P* < 0.05) and had greater disease duration compared to those with no to mild disability (*t* = 3.80, *P* < 0.05). The pw multiple sclerosis who had at least one PRL were younger (*t* = −2.05, *P* = 0.04) and had greater lesion volume compared to the pw multiple sclerosis without PRL (*t* = 7.61, *P* < 0.05). Thirty-six, 18 and 40 pw multiple sclerosis had impairments on SDMT, CVLT and BVMT, respectively. Twenty-one subjects were included in the CI group as these subjects showed impairments in at least two cognitive tests. Age and disease duration did not differ between CP and CI subjects (*t* = −0.89, *P* = 0.42 for age and *t* = −1.97, *P* = 0.08 for disease duration), while lesion volume was greater in pw multiple sclerosis with moderate to severe disability compared to those with no to mild disability and in CI compared to the CP group (*t* = 2.73, *P* = 0.02).

**Table 1:** Patient demographics and imaging features for all pw multiple sclerosis and the two sets of subgroups—no/mild versus moderate/severe disability and CP versus CI

	All pw multiple sclerosis (*n* = 482)	pw multiple sclerosis with no or mild disability (*n* = 379)	pw multiple sclerosis with moderate or severe disability (*n* = 103)	*P*-value (pw multiple sclerosis without versus with disability)	pw multiple sclerosis without PRL (*n* = 207)	pw multiple sclerosis with PRL (*n* = 156)	*P*-value (pw multiple sclerosis without versus with PRL)	CP (*n* = 150)	CI (*n* = 21)	*P*-value (CP versus CI)
Age	40 [33, 50]	38 [32, 47]	50 [40.15, 57.50]	<0.01	42.8 [35, 51.85]	39.5 [32.75, 49]	0.04	41.50 [35, 50.75]	44 [36, 53]	0.4228
Female %	71.57	73.08	66.01	0.24761	77.77	68.58	0.06	73.33	71.42	0.99
Disease duration	6 [2.56, 52]	5.49 [2.04, 11]	9.87 [3.88, 16.95]	0.00054	8.39 [3.9, 13.25]	7.08 [3.01, 13.08]	0.22	9.2 [4.625, 13.2]	13.50 [7.9, 17.6]	0.0837
EDSS	1.5 [0, 2.00]	1 [0, 2]	3.5 [3, 6]	<2.2e−16	1 [0, 2.5]	1.5 [0, 3]	0.17	1 [0, 2]	2 [2, 3.5]	0.00167
Lesion volume on T2 FLAIR (mm^3^)	3808 [1255, 9915]	3534 [1232, 8466]	6008 [2085, 20 198]	0.00017	2059 [756.5, 5931]	10 205 [4726, 19 410]	<2.2e−16	1995.5 [822.5, 5863.5]	9235 [2795, 17 295]	0.02117
SDMT								59 [51, 65]	36 [35, 44]	<2.2e−16
CVLT								55 [49, 61]	43 [39, 45]	<2.2e−16
BVMT								26 [23, 30]	17 [13, 30]	<2.2e−16

The *P*-values were obtained in the comparison of no/mild versus moderate/severe disability groups (fifth column) and CP versus CI (eighth column) using the Wilcoxon rank-sum test. Values were presented as median [first quartile, third quartile] for the continuous variables. *P*-values were corrected with the BH method for multiple comparisons. Age and disease duration were measured in years.

### Sequence of structural disconnection due to multiple sclerosis lesions across the spectrum of disability

**Figure 2:**
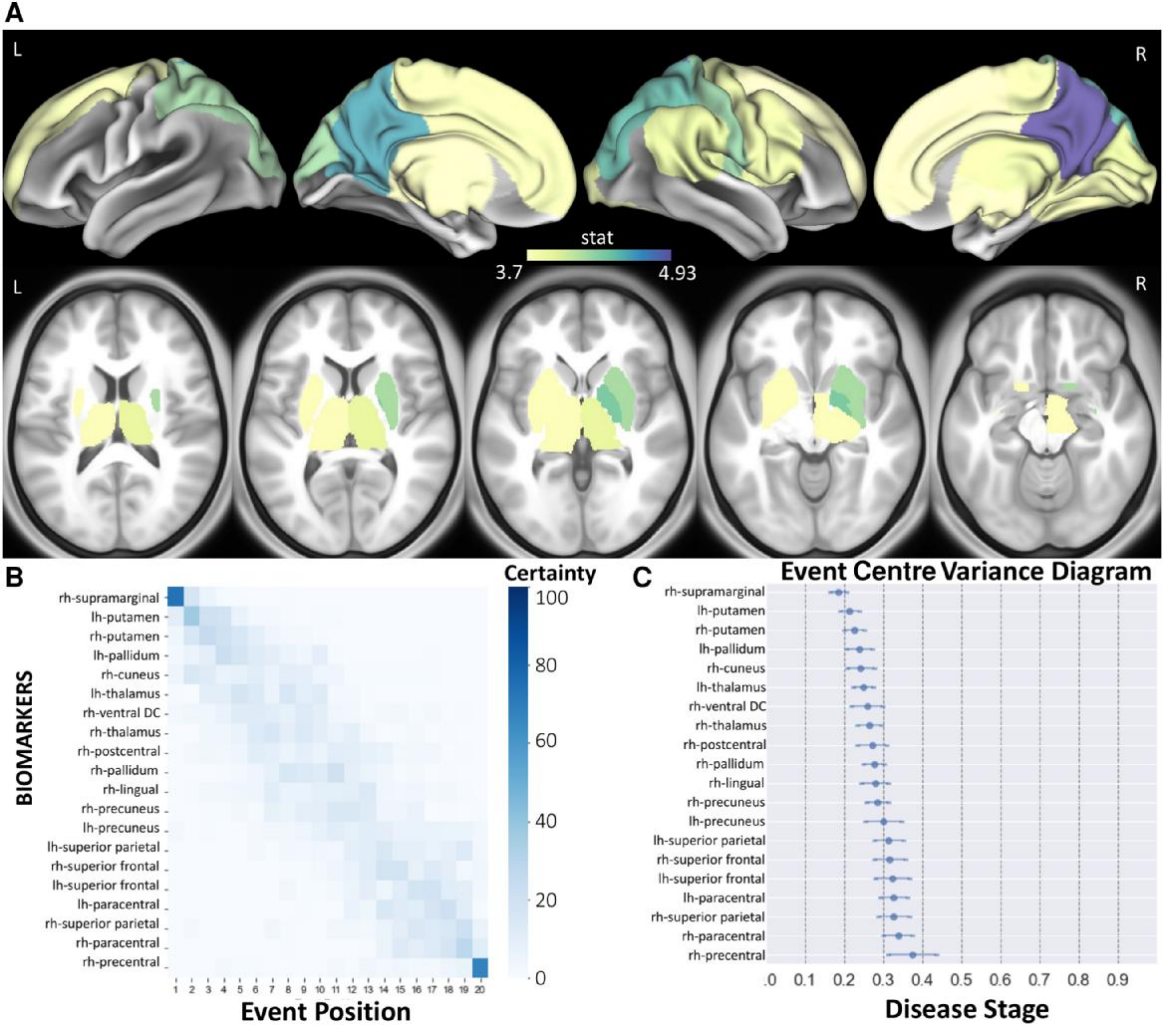
**The sequence of regional disconnectivity due to T2 FLAIR lesions across the spectrum of disability.** (**A**) Student’s *t*-statistic comparing the log of the T2 FLAIR lesion regional structural disconnectivity between disability groups, displaying the 20 regions having the largest *t*-statistics (positive *t*-statistic = structural disconnectivity is greater in individuals with moderate/severe disability). All subjects (*n* = 482) were used for this analysis. (**B**) The 20 regions having the largest *t*-statistics for each comparison (disability or cognition) are used in the dEBM. Positional variance diagram for disability progression for the same 20 regions. The maximum likelihood sequence of abnormality is shown on the *y*-axis (*top* to *bottom*). (**C**) The event centre variance diagram shows the standard error of estimated abnormality centres. rh/R, right hemisphere; lh/L, left hemisphere.

Figure 2 shows the *t*-statistics of the structural disconnectivity metrics for the 20 regions that were the most significantly different between disability groups and the dEBM model results, visualized as the sequence of the structural disconnectivity metrics across disability severity. All subjects (*n* = 482) were used for this analysis. The structural disconnectivity metrics were significantly greater, particularly in the bilateral precuneus, right postcentral and right superior parietal regions in the moderate/severe disability group compared to the no/mild disability group (Fig. 2A). The dEBM results showed that structural disconnectivity due to T2 FLAIR lesions occurs first in the right supramarginal, followed by subcortical regions including putamen, pallidum, cuneus and thalamus as disability becomes more severe (Fig. 2B and C). The sequence of the regional structural disconnectivity obtained from this model was found to be significantly different from random sequence assignment (*t* = 4.17, *P* = 0.0005).

### PRL and non-PRL maps and their subsequent disruptions to structural connections

**Figure 3:**
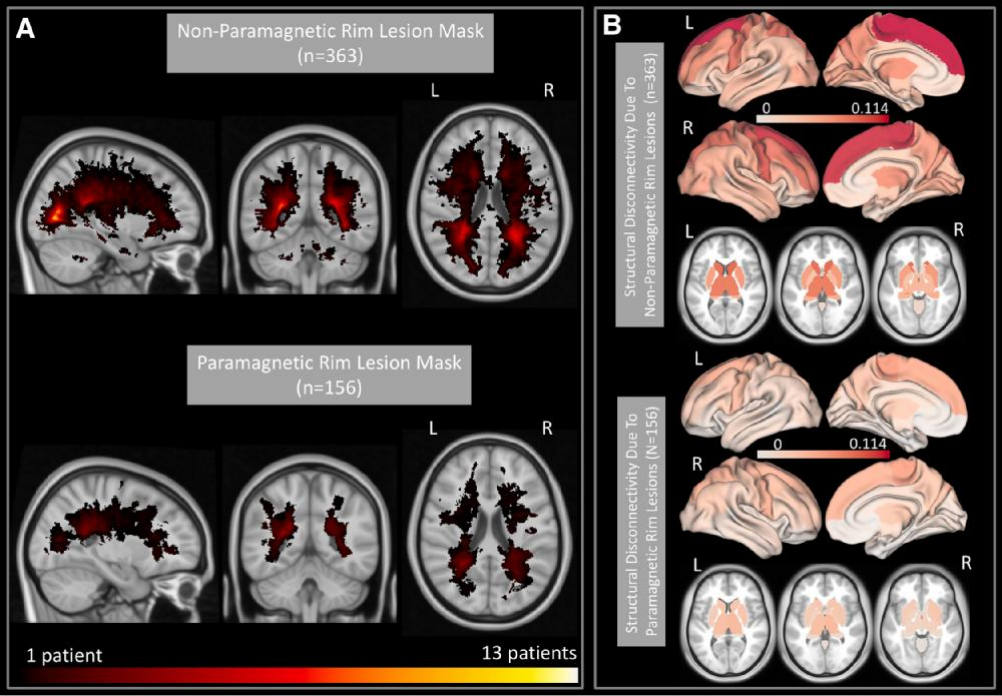
**PRL versus non-PRL masks and the regional structural disconnectivity due to PRL and non-PRL.** (**A**) A heat map of the lesion masks for the non-PRL (T2 FLAIR lesions minus PRL) in 363 subjects and the PRL in 156/363 subjects with ≥1 PRL. (**B**) Regional structural disconnectivity due to the non-PRL and PRL, averaged over their respective cohorts. Darker colours indicate higher structural disconnectivity in the region.

As mentioned above, only 363 out of 482 patients had QSM imaging and thus had separate lesion masks for PRL and non-PRL. Figure 3 shows the heat maps of lesion masks of non-PRL (*n* = 363) and PRL (*n* = 156 subjects with ≥1 PRL) and their resulting structural disconnectivity maps. Heat maps of the lesion masks show that PRL tended to cluster in periventricular WM (Fig. 3A), compared to non-PRLs that are more widespread throughout the WM (Fig. 3B). The structural disconnectivity due to non-PRL was greater compared to those due to PRL. Note the scale differences in the two modalities are primarily due to the relatively fewer PRL subjects as compared to non-PRL.

### Sequence of structural disconnection due to PRL and non-PRL across the spectrum of disability

**Figure 4:**
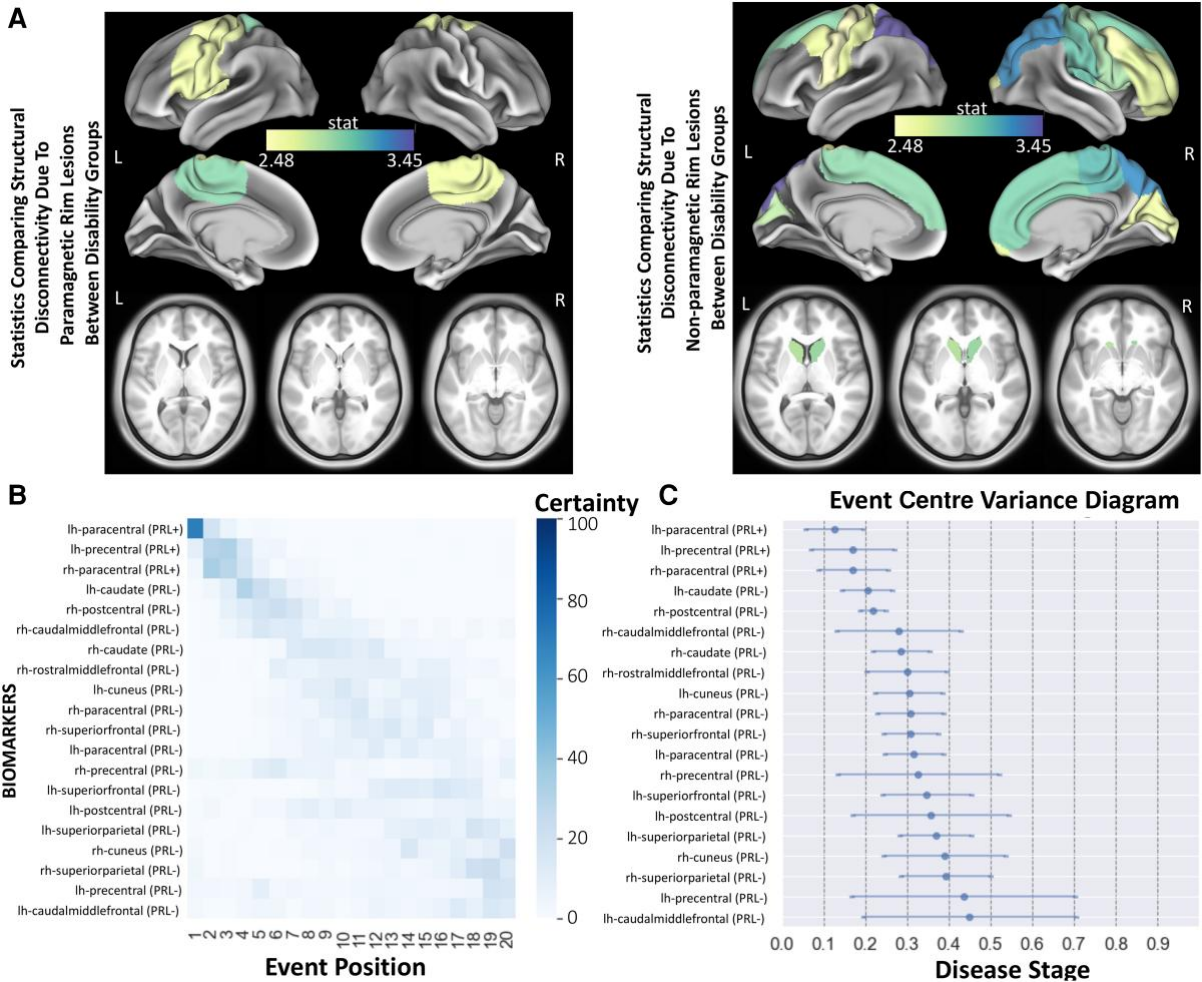
**The sequence of regional disconnectivity due to PRL and non-PRL across the spectrum of disability.** (**A**) Student’s *t*-statistic comparing the log of the regional structural disconnectivity between disability groups; only the 20 regions having the largest *t*-statistics across both non-PRL and PRL masks are shown. The subjects who had QSM imaging and thus had separate lesion masks for PRL and non-PRL (*n* = 363) were used for this analysis. (**B**) The 20 regions having the largest *t*-statistics for each comparison (disability or cognition) are used in the dEBM. Positional variance diagram across disability progression for the same 20 regions presented in **A**. The maximum likelihood sequence of abnormality is shown on the *y*-axis (*top* to *bottom*). Colour intensity in each row indicates positional variance: the darker the colour, the higher the confidence of the event position across 100 bootstraps (i.e. certainty). (**C**) The event centre variance diagram shows the standard error of estimated abnormality centres. rh/R, right hemisphere; lh/L, left hemisphere.

We compared the regional structural disconnectivity due to non-PRL or PRL (86 regions × 2 lesion mask types = 172 structural disconnectivity metrics in total) between disability groups in subjects who had QSM imaging and thus had separate lesion masks for PRL and non-PRL (*n* = 363). The 20 regions where the structural disconnectivity was most significantly different (out of these 172 metrics) are presented in Fig. 4A and B. The structural disconnectivity metrics were greater in the moderate/severe disability group compared to no/mild disability group across all 20 regions. Only 3/20 of the top metrics were structural disconnections due to PRL. The structural disconnections that were the most significantly different between disability groups were mostly located in the frontal, motor-related and subcortical regions (Fig. 4A and B). The structural disconnectivity due to PRL and non-PRL in the precentral, postcentral and paracentral regions was commonly found as greater in the moderate/severe disability group compared to the no/mild disability group. Further, the dEBM results showed that structural disconnectivity due to PRL in the bilateral paracentral gyrus and left precentral gyrus was found to occur first, followed by the structural disconnectivity due to non-PRL in the bilateral caudate, right postcentral gyrus and right caudal middle frontal gyrus. The sequence of the regional structural disconnectivity obtained from this model was found to be significantly different than random sequence assignment (*t* = 3.62, *P* = 0.001).

### Sequence of structural disconnection due to multiple sclerosis lesions across the spectrum of cognitive impairment

**Figure 5:**
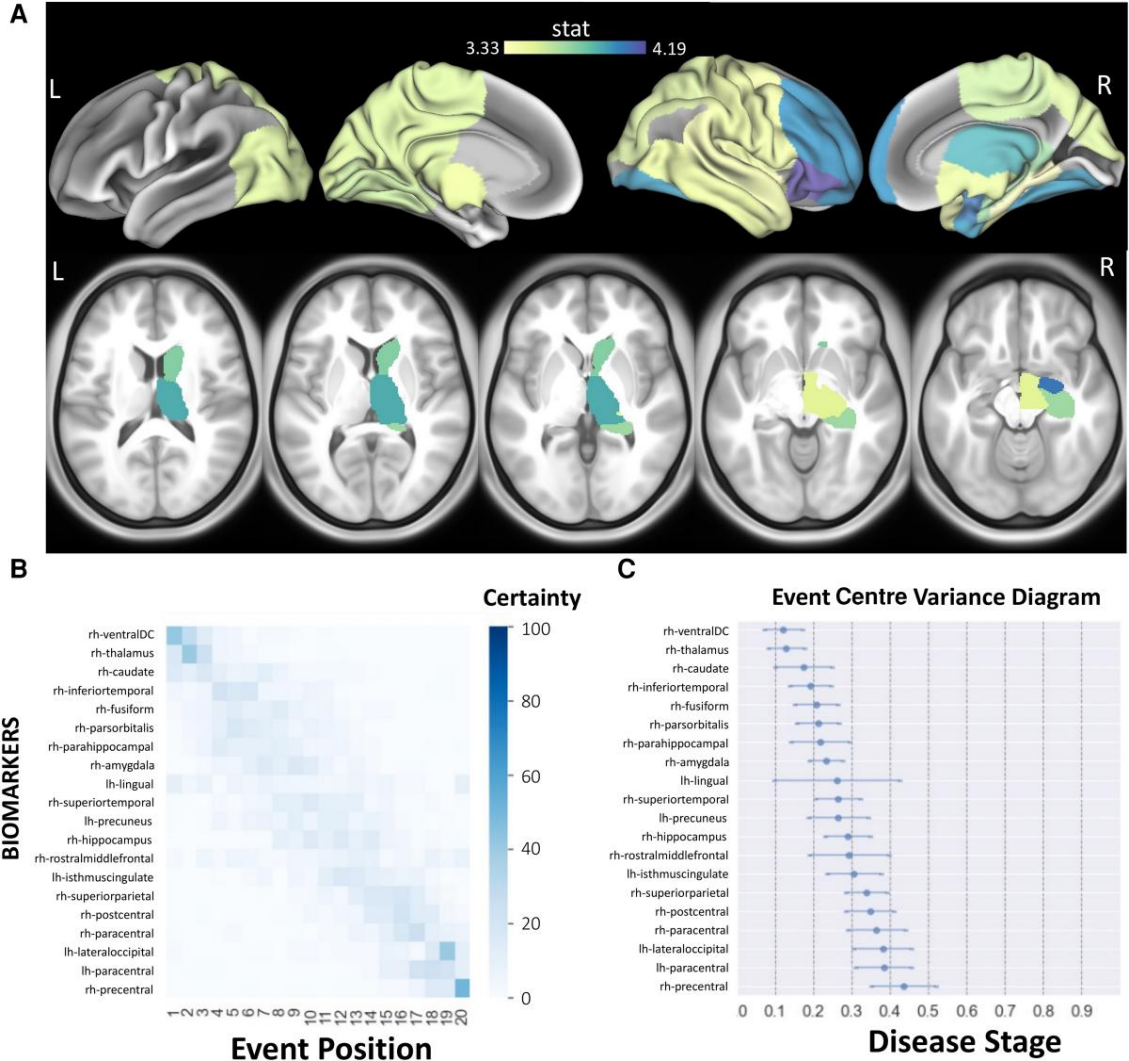
**The sequence of regional disconnectivity due to T2 FLAIR lesions across the spectrum of cognitive impairment.** (**A**) Student’s *t*-statistic comparing the log of the T2 FLAIR lesion regional structural disconnectivity between CP and CI groups. The subjects who had cognitive scores (*n* = 171) were used for this analysis. Darker colours indicate greater *t*-statistics comparing the structural disconnectivity in the CI group compared to the CP group. (**B**) The 20 regions having the largest *t*-statistics for each comparison (disability or cognition) are used in the dEBM. Positional variance diagram across cognitive impairment severity for the same 20 regions presented in **A**. The maximum likelihood sequence of abnormality is shown on the *y*-axis (*top* to *bottom*). Colour intensity in each row indicates positional variance: the darker the colour, the higher the confidence of the event position across 100 bootstraps (i.e. certainty). (**C**) The event centre variance diagram shows the standard error of estimated abnormality centres. rh/R, right hemisphere; lh/L, left hemisphere.

Figure 5 shows the *t*-statistics of the regional (T2 FLAIR lesion-based) structural disconnectivity for the 20 most significantly different regions between CI and CP, as well as the sequence of the regional structural disconnectivity metrics across cognitive impairment. The subjects who had cognitive scores (*n* = 171) were used for this analysis. In all 20 regions, the structural disconnectivity was significantly higher in the CI group compared to the CP group (corrected *P* < 0.05) and was most different in the right subcortical and frontal regions. The dEBM results show that structural disconnectivity in right hemisphere subcortical regions, particularly in the right ventral diencephalon (DC), thalamus and caudate, occurs earlier across cognitive impairment severity. The sequence of the regional structural disconnectivity obtained from this model was found to be significantly different than random sequence assignment (*t* = 4.81, *P* = 0.0001).

As mentioned in the ‘Materials and methods’ section, patients were assigned the CI group when at least two out of three cognitive tests were identified as below normal. As a *post hoc* analysis, we reduced this requirement to only one out of three cognitive tests being abnormal for assignment to the CI group. We found similar results wherein the structural disconnectivity in subcortical and frontal, along with visual regions, occurs in the earlier stages across cognitive impairment severity (see Supplementary Fig. 2).

### Sequence of structural disconnection due to PRL and non-PRL across the spectrum of cognitive impairment

We also investigated the sequence of PRL and non–PRL-based structural disconnectivity across cognitive impairment severity in all patients with cognitive scores (*n* = 171). Our results showed that structural disconnectivity due to non-PRL occurs at earlier stages compared to structural disconnectivity due to PRL across cognitive impairment severity. Similar to our results with T2 FLAIR lesions, non-PRL structural disconnectivity in subcortical regions, particularly in the right ventral DC, caudate and thalamus, occurs early in the event sequence (see Supplementary Fig. 3).

## Discussion

In this study, we used dEBM applied to cross-sectional data in pw multiple sclerosis to investigate the sequence of regional structural disconnectivity across the spectrum of disability and cognitive impairment. We computed structural disconnectivity metrics using the NeMo tool and overall T2 FLAIR lesions as well as these lesions separated into QSM hyperintense paramagnetic rim (PRL) or no rim (non-PRL). We showed that structural disconnectivity due to T2 FLAIR lesions in the ventral attention and subcortical networks, in particular within the supramarginal and putamen, occurs earlier in the sequence of disability (i.e. mild to severe). We also showed that PRL-based structural disconnectivity in motor-related regions occurs earlier in the disability event sequence, followed by non-PRL-based structural disconnectivity in the caudate and postcentral gyrus. T2 FLAIR lesion-based structural disconnectivity in subcortical regions, including the thalamus, occurs earliest in the sequence of cognitive impairment (i.e. preserved to impaired).

### Structural disconnectivity in subcortical regions is central to disability and cognitive impairment

The distribution of multiple sclerosis lesions is not uniform across the brain. Histopathological studies have shown that multiple sclerosis lesions tend to more frequently appear in periventricular, juxtacortical and subpial locations^1^; however, lesion location and size are quite heterogeneous, which contributes to the widely varying physical and cognitive impairment among pw multiple sclerosis.^30-33^

Subcortical regions have been shown to play a large role in multiple sclerosis, as atrophy in this region has been associated with motor and cognitive impairment as well as fatigue.^34,35^ In alignment with these findings, one of our recent studies demonstrated that increased structural disconnectivity due to PRL and non-PRL in the thalamus was associated with greater disability in pw multiple sclerosis.^5^ Moreover, other studies have shown that atrophy in subcortical regions, particularly in the thalamus, pallidum, putamen and cuneus, occurs as an early event in relation to disability progression in multiple sclerosis.^2,3^ These results are consistent with the findings of our study that uses structural disconnectivity metrics, which were previously shown to be associated with atrophy in certain subcortical regions.^36^

Only one study investigated the sequence of regional/network-based structural and functional changes across cognitive impairment severity in multiple sclerosis.^3^ This study used a different cognitive test than our study (i.e. Brief Repeatable Battery of Neuropsychological Tests) to assign subjects into CI, mildly CI and CP groups and investigated the sequence of the events across the spectrum of cognitive impairment. Thalamic atrophy was found to occur second, just after increased functional centrality in the default mode network, as cognitive impairment became more severe. This is in concordance with our results that show structural disconnection in the right hypothalamus (ventral DC) and right thalamus occurs earliest across the spectrum of cognitive impairment. Thalamic lesions are very common in multiple sclerosis; 71% of patients have thalamic lesions on 7 T MRI^37^ and larger thalamic lesions have been associated with greater cognitive impairment.^38^ Altogether, these results support the commonly reported finding that damage to the thalamus, either in GM or connected WM, plays an important role in both physical and cognitive impairments in multiple sclerosis.

### Structural disconnectivity due to PRL occurs earlier in the spectrum of disability severity

While the impact of lesion location on disability type/severity has been previously studied in multiple sclerosis,^5,30,39^ there are fewer studies that attend to the role of chronic lesion subtype, i.e. chronic active (PRL) versus inactive (non-PRL). Our previous study found that structural disconnectivity from PRL more accurately predicted disability compared to structural disconnectivity from non-PRL.^5^ Moreover, increased PRL-based structural disconnectivity in the thalamus and cerebellum was commonly found to be associated with worse disability. However, the sequence of PRL versus non-PRL resulting structural disconnections across disability severity had not been studied. Our study shows that structural disconnectivity due to PRL, particularly in motor-related regions such as bilateral paracentral and left precentral, occurs earlier across the spectrum of disability severity. It may be for this reason that chronic active lesions, although the far minority of lesions, demonstrate an association with clinical disability,^40,41^ and these results suggest that they may be an early biomarker of disease progression. It has been previously shown that the neurodegeneration due to multiple sclerosis lesions may impact both the axonal terminal and the cell soma, which may then lead to atrophy in the connected GM regions.^42^ WM lesion volume was shown to be associated with GM atrophy at both global and regional levels.^43-45^ Thus, the structural disconnectivity due to PRL in the motor-related regions might cause atrophy in these regions. Our findings align with previous evidence that physical dysfunction and atrophy in the sensorimotor cortex are related.^46-49^ Interestingly, PRL did not have the same relevance when evaluating cognitive impairment as compared to EDSS, and we suspect this difference may relate to relatively small number of CI patients in combination with a relatively rare occurrence of a PRL. PRLs have been associated with cognitive impairment^40,41^; thus, this should be repeated in a larger sample of patients with cognitive testing.

### dEBM in pw multiple sclerosis

dEBM allows investigation of the sequence of biomarker abnormality across disability/impairment progression using large cross-sectional neuroimaging data sets, which are easier to collect than longitudinal data. This approach has been applied to data from pw multiple sclerosis to investigate the sequence of regional atrophy^2^ as well as structural, functional and cognitive changes.^3^ The latter study used structural connectivity measured with dMRI,^3^ which stands in contrast to our approach that estimates the structural disconnection from the lesion masks and the NeMo Tool. In addition to its wider clinical applicability, our approach to measure structural disconnectivity has also been shown to be a better predictor of disability compared to structural and functional connectivity metrics computed with advanced imaging techniques.^8^ Lastly, our study did not use *a priori*–selected regions but rather included all cortical, subcortical and cerebellar regions before statistical selection of the regions to include in the dEBM. This data-driven approach is advantageous as it is informed by the computationally intensive use of the existing data set and therefore provides less biased conclusions from the data compared to hypothesis-driven approach.

### Limitations

The lesion location and sequence of the lesion appearance are very heterogeneous among multiple sclerosis patients. As such, the existence of a meaningful sequence of lesion-related structural disconnections that holds consistently across subjects remains uncertain. In our study, we showed that the sequence of the regional structural disconnectivity is significantly different from a random sequence. However, to enhance the interpretation of the results, future studies could benefit from discussing the null hypothesis explicitly and conducting statistical tests to provide a more in-depth analysis of the overlap and variability between the sequence of lesion-related structural disconnectivity to assess the reliability and meaningfulness of the observed sequences. Another limitation of our study is that the regional structural disconnectivity metrics were computed by considering only the lesion size and location. As various pathologies such as myelin and/or axonal content cannot be discerned on T2 FLAIR, the differential impact of specific lesions’ pathologies on structural connections was not considered. In addition, the regional sequence of the structural disconnections was not investigated for different phenotypes of multiple sclerosis such as CIS, relapsing remittent multiple sclerosis (RRMS), secondary progressive multiple sclerosis (SPMS), and primary progressive multiple sclerosis (PPMS). A future study that could investigate if the sequence of the structural disconnections differs for different phenotypes might be beneficial; however, traditional clinical classifiers may not be relevant.^50^ Presence or absence of treatment and type of treatments likely contribute to disability progression and the appearance of new lesions; this information was not included in the dEBM model. A study that includes a similar amount of patients in each treatment category should be performed. Additionally, while cross-sectional data provide valuable insights into a specific point in time, they may not capture the dynamic nature of certain conditions. It is important to note that the appearance of PRLs is temporary, as they can become non-PRL over time, reflecting the fluctuating nature of the inflammatory process in the CNS. Therefore, these results need to be replicated with a longitudinal data set to have a more nuanced understanding of the temporal dynamics of PRLs and clinical implications of these lesions. It should be emphasized that while dEBM provides an order of the events (i.e. a region’s structural disconnection) relative to one another, the time between the events or the exact timing of the event cannot be identified due to non-linear appearance of the events and utilization of a cross-sectional data. Future implementations of this model need to incorporate longitudinal data to assess the temporal relationship between disability and regional structural disconnectivity patterns. Finally, the control subjects in the NeMo Tool ranged mostly from 21 to 35, which is younger than many of the pw multiple sclerosis in our study; any aging effects on WM or structural connectivity in the pw multiple sclerosis would not be considered in the structural disconnection metrics.

## Conclusion

This is the first study to investigate the sequence of lesion-based regional structural disconnectivity across the spectrum of disability and cognitive impairment severities in pw multiple sclerosis. We found that structural disconnections in subcortical regions, including the thalamus, occur earlier in the spectrum of disability and cognitive impairment. We also found that PRL-based structural disconnectivity in motor-related regions occurs earliest in disability progression. This study provides a better understanding of how structural disconnectivity due to T2 FLAIR lesions and PRL/non-PRL deferentially impacts disability and cognitive impairment in multiple sclerosis. A deeper understanding of the regional pattern of structural disconnection due to different types of lesions may help in identifying the early biomarkers of disability, creating more accurate prognoses and perhaps personalize treatment selection to reduce the burden of multiple sclerosis.

## Supplementary Material

fcad332_Supplementary_Data

## Data Availability

The deidentified data that support the findings of this study are available upon reasonable request from the corresponding author. The codes that were used to perform the dEBM analysis are publicly available (https://github.com/EuroPOND/pyebm).

## References

[1] Brownell B , Hughes JT. The distribution of plaques in the cerebrum in multiple sclerosis. J Neurol Neurosurg Psychiatry. 1962;25(4):315–320. 14016083 10.1136/jnnp.25.4.315 PMC495470

[2] Eshaghi A , Marinescu RV, Young AL, et al Progression of regional grey matter atrophy in multiple sclerosis. Brain. 2018;141(6):1665–1677. 29741648 10.1093/brain/awy088 PMC5995197

[3] Dekker I , Schoonheim MM, Venkatraghavan V, et al The sequence of structural, functional and cognitive changes in multiple sclerosis. Neuroimage Clin. 2021;29:102550. 33418173 10.1016/j.nicl.2020.102550 PMC7804841

[4] Kuceyeski A , Monohan E, Morris E, Fujimoto K, Vargas W, Gauthier SA. Baseline biomarkers of connectome disruption and atrophy predict future processing speed in early multiple sclerosis. Neuroimage Clin. 2018;19:417–424. 30013921 10.1016/j.nicl.2018.05.003 PMC6019863

[5] Tozlu C , Jamison K, Nguyen T, et al Structural disconnectivity from paramagnetic rim lesions is related to disability in multiple sclerosis. Brain Behav. 2021;11(10):e2353. 34498432 10.1002/brb3.2353 PMC8553317

[6] Kuceyeski A , Maruta J, Relkin N, Raj A. The Network Modification (NeMo) Tool: Elucidating the effect of white matter integrity changes on cortical and subcortical structural connectivity. Brain Connect. 2013;3(5):451–463. 23855491 10.1089/brain.2013.0147 PMC3796322

[7] Kuceyeski A , Navi BB, Kamel H, et al Exploring the brain’s structural connectome: A quantitative stroke lesion-dysfunction mapping study. Hum Brain Mapp. 2015;36(6):2147–2160. 25655204 10.1002/hbm.22761 PMC4414746

[8] Tozlu C , Jamison K, Gu Z, Gauthier SA, Kuceyeski A. Estimated connectivity networks outperform observed connectivity networks when classifying people with multiple sclerosis into disability groups. Neuroimage Clin. 2021;32:102827. 34601310 10.1016/j.nicl.2021.102827 PMC8488753

[9] Fuchs TA , Dwyer MG, Kuceyeski A, et al White matter tract network disruption explains reduced conscientiousness in multiple sclerosis. Hum Brain Mapp. 2018;39(9):3682–3690. 29740964 10.1002/hbm.24203 PMC6866584

[10] Fuchs TA , Ziccardi S, Benedict RHB, et al Functional connectivity and structural disruption in the default-mode network predicts cognitive rehabilitation outcomes in multiple sclerosis. J Neuroimaging. 2020;30(4):523–530. 32391981 10.1111/jon.12723

[11] Dal-Bianco A , Grabner G, Kronnerwetter C, et al Slow expansion of multiple sclerosis iron rim lesions: Pathology and 7 T magnetic resonance imaging. Acta Neuropathol. 2017;133(1):25–42. 27796537 10.1007/s00401-016-1636-z PMC5209400

[12] Absinta M , Sati P, Schindler M, et al Persistent 7-tesla phase rim predicts poor outcome in new multiple sclerosis patient lesions. J Clin Invest. 2016;126(7):2597–2609. 27270171 10.1172/JCI86198 PMC4922708

[13] Dal-Bianco A , Grabner G, Kronnerwetter C, et al Long-term evolution of multiple sclerosis iron rim lesions in 7 T MRI. Brain. 2021;144(3):833–847. 33484118 10.1093/brain/awaa436

[14] de Rochefort L , Liu T, Kressler B, et al Quantitative susceptibility map reconstruction from MR phase data using Bayesian regularization: Validation and application to brain imaging. Magn Reson Med. 2010;63(1):194–206. 19953507 10.1002/mrm.22187

[15] Deh K , Nguyen TD, Eskreis-Winkler S, et al Reproducibility of quantitative susceptibility mapping in the brain at two field strengths from two vendors. J Magn Reson Imaging. 2015;42(6):1592–1600. 25960320 10.1002/jmri.24943 PMC4661140

[16] Kaunzner UW , Kang Y, Zhang S, et al Quantitative susceptibility mapping identifies inflammation in a subset of chronic multiple sclerosis lesions. Brain. 2019;142(1):133–145. 30561514 10.1093/brain/awy296 PMC6308309

[17] Chen W , Gauthier SA, Gupta A, et al Quantitative susceptibility mapping of multiple sclerosis lesions at various ages. Radiology. 2014;271(1):183–192. 24475808 10.1148/radiol.13130353 PMC4263629

[18] Yao Y , Nguyen TD, Pandya S, et al Combining quantitative susceptibility mapping with automatic zero reference (QSM0) and myelin water fraction imaging to quantify iron-related myelin damage in chronic active MS lesions. AJNR Am J Neuroradiol. 2018;39(2):303–310. 29242359 10.3174/ajnr.A5482 PMC5812818

[19] Zhang S , Nguyen TD, Ruá SMH, et al Quantitative susceptibility mapping of time-dependent susceptibility changes in multiple sclerosis lesions. Am J Neuroradiol. 2019;40(6):987–993. 31097429 10.3174/ajnr.A6071 PMC6565472

[20] Kurtzke JF . Rating neurologic impairment in multiple sclerosis: An expanded disability status scale (EDSS). Neurology. 1983;33(11):1444–1452. 6685237 10.1212/wnl.33.11.1444

[21] Langdon DW , Amato MP, Boringa J, et al Recommendations for a brief international cognitive assessment for multiple sclerosis (BICAMS). Mult Scler. 2012;18(6):891–898. 22190573 10.1177/1352458511431076 PMC3546642

[22] de Meo E , Portaccio E, Giorgio A, et al Identifying the distinct cognitive phenotypes in multiple sclerosis. JAMA Neurol. 2021;78(4):414–425. 33393981 10.1001/jamaneurol.2020.4920 PMC7783596

[23] Zinger N , Ponath G, Sweeney E, et al Dimethyl fumarate reduces inflammation in chronic active multiple sclerosis lesions. Neurol Neuroimmunol Neuroinflamm. 2022;9(2):e1138. 35046083 10.1212/NXI.0000000000001138 PMC8771666

[24] Spincemaille P , Liu Z, Zhang S, et al Clinical integration of automated processing for brain quantitative susceptibility mapping: Multi-site reproducibility and single-site robustness. J Neuroimaging. 2019;29(6):689–698. 31379055 10.1111/jon.12658 PMC6814493

[25] Jenkinson M , Bannister P, Brady M, Smith S. Improved optimization for the robust and accurate linear registration and motion correction of brain images. Neuroimage. 2002;17(2):825–841. 12377157 10.1016/s1053-8119(02)91132-8

[26] Fischl B , Sereno MI, Dale AM. Cortical surface-based analysis: II. Inflation, flattening, and a surface-based coordinate system. Neuroimage. 1999;9(2):195–207. 9931269 10.1006/nimg.1998.0396

[27] Fonteijn HM , Modat M, Clarkson MJ, et al An event-based model for disease progression and its application in familial Alzheimer’s disease and Huntington’s disease. Neuroimage. 2012;60(3):1880–1889. 22281676 10.1016/j.neuroimage.2012.01.062

[28] Venkatraghavan V , Bron EE, Niessen WJ, Klein S. Disease progression timeline estimation for Alzheimer’s disease using discriminative event based modeling. Neuroimage. 2019;186:518–532. 30471388 10.1016/j.neuroimage.2018.11.024

[29] Benjamini Y , Hochberg Y. Controlling the false discovery rate: A practical and powerful approach to multiple testing. J R Stat Soc Seri B (Methodol). 1995;57(1):289–300.

[30] Krieger SC , Lublin FD. Location, location, location. Mult Scler. 2018;24(11):1396–1398. 30047828 10.1177/1352458518790385

[31] Pardini M , Sudre CH, Prados F, et al Relationship of grey and white matter abnormalities with distance from the surface of the brain in multiple sclerosis. J Neurol Neurosurg Psychiatry. 2016;87(11):1212–1217. 27601434 10.1136/jnnp-2016-313979

[32] Mainero C , Louapre C, Govindarajan S, et al A gradient in cortical pathology in multiple sclerosis by in vivo quantitative 7 T imaging. Brain. 2015;138:932–945. 25681411 10.1093/brain/awv011 PMC4677339

[33] Nowaczyk N , Kalinowska-ŁYszczarz A, Paprzycki W, Michalak S, Kaźmierski R, Pawlak MA. Spatial distribution of white matter degenerative lesions and cognitive dysfunction in relapsing-remitting multiple sclerosis patients. Neurol Neurochir Pol. 2019;53(1):18–25. 30742302 10.5603/PJNNS.a2018.0001 PMC8800152

[34] Nourbakhsh B , Azevedo C, Maghzi AH, Spain R, Pelletier D, Waubant E. Subcortical grey matter volumes predict subsequent walking function in early multiple sclerosis. J Neurol Sci. 2016;366:229–233. 27288812 10.1016/j.jns.2016.04.054

[35] Ramasamy DP , Benedict RHB, Cox JL, et al Extent of cerebellum, subcortical and cortical atrophy in patients with MS: A case-control study. J Neurol Sci. 2009;282(1-2):47–54. 19201003 10.1016/j.jns.2008.12.034

[36] Kuceyeski AF , Vargas W, Dayan M, et al Modeling the relationship among gray matter atrophy, abnormalities in connecting white matter, and cognitive performance in early multiple sclerosis. Am J Neuroradiol. 2015;36(4):702–709. 25414004 10.3174/ajnr.A4165 PMC4951088

[37] Harrison DM , Oh J, Roy S, et al Thalamic lesions in multiple sclerosis by 7T MRI: Clinical implications and relationship to cortical pathology. Mult Scler. 2015;21(9):1139–1150. 25583851 10.1177/1352458514558134 PMC4499502

[38] Mehndiratta A , Treaba CA, Barletta V, et al Characterization of thalamic lesions and their correlates in multiple sclerosis by ultra-high-field MRI. Mult Scler. 2021;27(5):674–683. 32584159 10.1177/1352458520932804 PMC7759586

[39] Guo Z , Long L, Qiu W, et al The distributional characteristics of multiple sclerosis lesions on quantitative susceptibility mapping and their correlation with clinical severity. Front Neurol. 2021;12:647519. 34305779 10.3389/fneur.2021.647519 PMC8299522

[40] Absinta M , Sati P, Masuzzo F, et al Association of chronic active multiple sclerosis lesions with disability in vivo. JAMA Neurol. 2019;76(12):1474–1483. 31403674 10.1001/jamaneurol.2019.2399 PMC6692692

[41] Marcille M , Hurtado Rúa S, Tyshkov C, et al Disease correlates of rim lesions on quantitative susceptibility mapping in multiple sclerosis. Sci Rep. 2022;12(1):4411. 35292734 10.1038/s41598-022-08477-6 PMC8924224

[42] Dendrou CA , Fugger L, Friese MA. Immunopathology of multiple sclerosis. Nat Rev Immunol. 2015;15(9):545–558. 26250739 10.1038/nri3871

[43] Lie IA , Weeda MM, Mattiesing RM, et al Relationship between white matter lesions and gray matter atrophy in multiple sclerosis. Neurology. 2022;98(15):e1562–e1573. 35173016 10.1212/WNL.0000000000200006 PMC9038199

[44] Chard DT , Miller DH. What lies beneath grey matter atrophy in multiple sclerosis? Brain. 2016;139(1):7–10. 26747854 10.1093/brain/awv354

[45] Steenwijk MD , Daams M, Pouwels PJW, et al What explains gray matter atrophy in long-standing multiple sclerosis? Radiology. 2014;272(3):832–842. 24761837 10.1148/radiol.14132708

[46] Sailer M , Fischl B, Salat D, et al Focal thinning of the cerebral cortex in multiple sclerosis. Brain. 2003;126(Pt 8):1734–1744. 12805100 10.1093/brain/awg175

[47] Bodini B , Khaleeli Z, Cercignani M, Miller DH, Thompson AJ, Ciccarelli O. Exploring the relationship between white matter and gray matter damage in early primary progressive multiple sclerosis: An in vivo study with TBSS and VBM. Hum Brain Mapp. 2009;30(9):2852–2861. 19172648 10.1002/hbm.20713 PMC6871131

[48] Prinster A , Quarantelli M, Lanzillo R, et al A voxel-based morphometry study of disease severity correlates in relapsing–remitting multiple sclerosis. Mult Scler. 2010;16(1):45–54. 20028706 10.1177/1352458509351896 PMC2841518

[49] Narayana PA , Govindarajan KA, Goel P, et al Regional cortical thickness in relapsing remitting multiple sclerosis: A multi-center study. Neuroimage Clin. 2012;2(1):120–131. 24179765 10.1016/j.nicl.2012.11.009 PMC3777814

[50] Kuhlmann T , Moccia M, Coetzee T, et al Multiple sclerosis progression: Time for a new mechanism-driven framework. Lancet Neurol. 2023;22(1):78–88. 36410373 10.1016/S1474-4422(22)00289-7 PMC10463558

