# Supplementary material for: The sequence of regional structural disconnectivity due to multiple sclerosis lesions
Source: Brain Commun. 2023 Dec 5;5(6):fcad332. doi: 10.1093/braincomms/fcad332 (PMC10724045; doi:10.1093/braincomms/fcad332)
Supplement: fcad332_Supplementary_Data [file fcad332_supplementary_data.pdf]

The sequence of regional structural disconnectivity due to multiple sclerosis lesions

SUPPLEMENTARY MATERIALS

Network Modification Tool

The Network Modification 2.0 (NeMo 2.0) tool estimates structural connectivity disruption due to a lesion mask using a database of healthy controls' structural connectivity information. The NeMo Tool uses a database of whole-brain tractograms from 420 unrelated healthy controls (206 female, 214 male, 28.7 $\pm$ 3.7 years) from the Human Connectome Project Young Adult (HCP-YA) dataset. The HCP diffusion data has 1.25 mm isotropic voxels, 3 shells (b=1000, 2000 and 3000) with 90 directions per shell, and was collected with both R-L and L-R phase encoding. HCP data have been minimally preprocessed to correct for motion, EPI and eddy-current distortion, and registered to subject T1 anatomy[1]. We used MRtrix3 to estimate a voxel-wise multi-shell, multi-tissue constrained spherical deconvolution model[2], followed by whole-brain deterministic (sd-stream) tractography with MRtrix3[3] with dynamic white-matter seeding[4]. Streamlines for each HCP subject were warped into a common volumetric space (MNI152) to create the final reference set. Given a lesion mask in MNI152 space, the NeMo tool identifies all streamlines that pass through the lesioned voxels. The NeMo Tool computes an estimated regional structural disconnectivity score for each gray matter region in a given atlas, representing the fraction of streamlines connecting those pairs of regions or connecting to that region that pass through the lesion.

The number of the pwMS used for each analysis

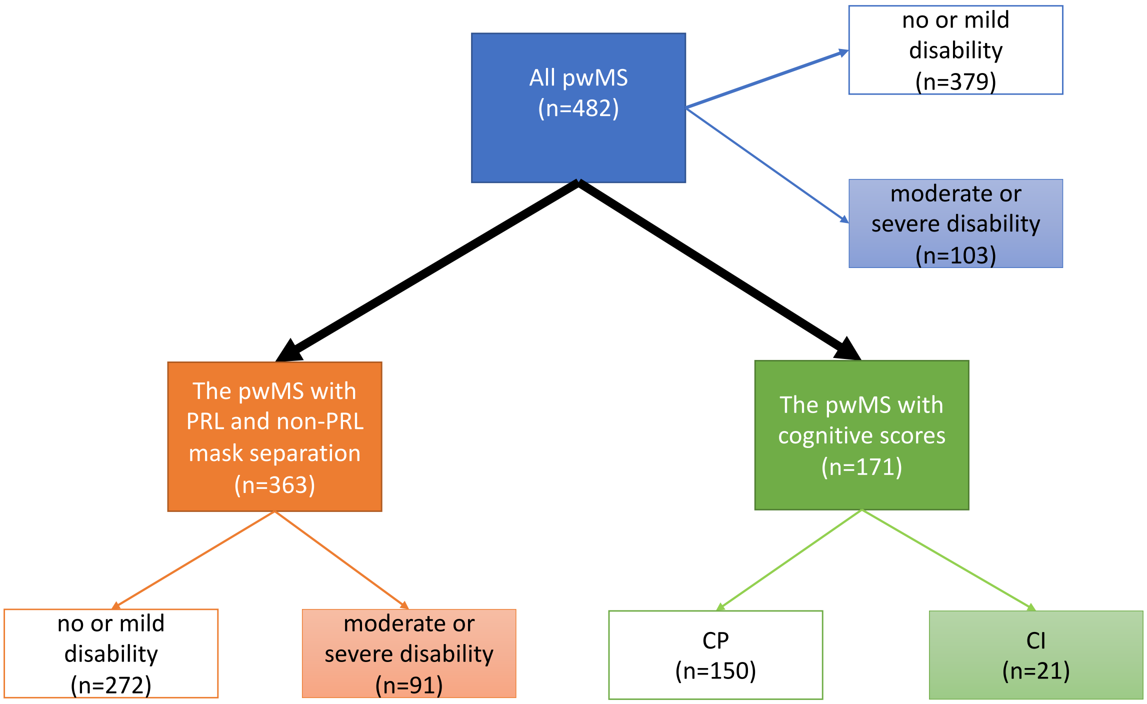

Supplementary Figure 1. We used the T2 FLAIR lesion masks of 482 pwMS to estimate the regional structural disconnectivity matrices. One hundred and three of 482 pwMS had an EDSS $\geq$ 3 (in the moderate to severe disability group). Only 363/482 pwMS had QSM imaging and thus the PRL/non-PRL labeling for their T2 FLAIR lesion masks. Ninety-one of 363 pwMS had an EDSS $\geq$ 3 in the moderate to severe disability group. We had the cognitive scores for a subset of the pwMS (N=171). Twenty-one of 171 pwMS were identified as CI (i.e. identified as CI in at least 2 tests out of 3 cognitive tests).

DEBM results when defining CI more liberally and using T2 FLAIR lesion dysconnectivity

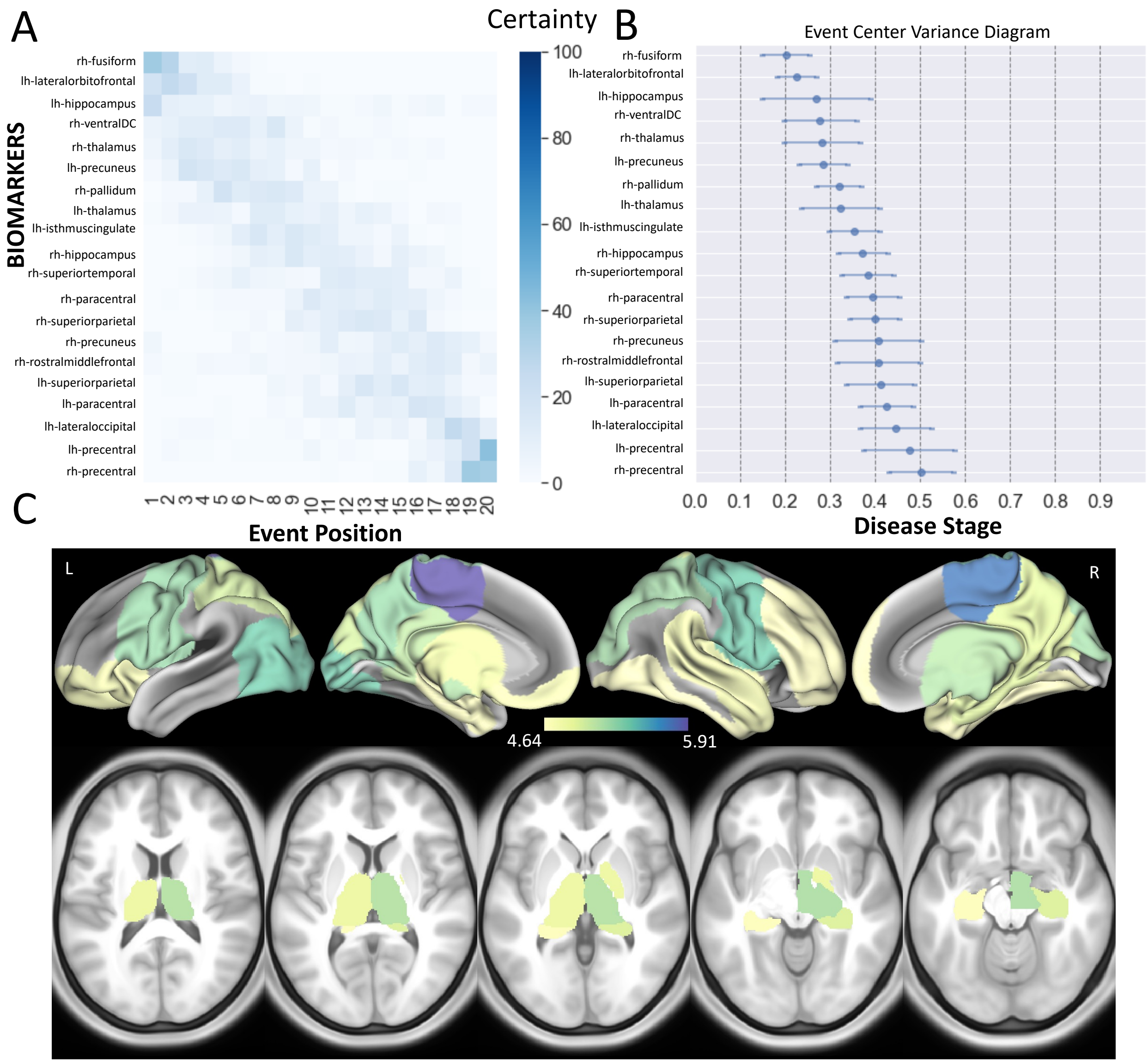

Supplementary Figure 2. (A) Positional variance diagram for cognitive impairment progression in pwMS for the 20 regions in which the disconnectivity metrics were the most significantly different between CP and CI, where the CI group is identified as being impaired in at least 1 out of 3 cognitive tests. The maximum-likelihood sequence of abnormality is shown on the y-axis (top to bottom). Color intensity in each row indicates positional variance: the darker the color, the higher

the confidence of the event position across 100 bootstraps. (B) The event-center variance diagram showing the standard error of estimated abnormality centers. (C) The statistics of the Student's t-test performed to compare the logarithm of the T2 FLAIR lesion-based regional structural disconnectivity between cognition groups; the 20 regions with largest t-statistics are shown. rh/R= right hemisphere and lh/L=left hemisphere.

DEBM results when defining CI more liberally and using PRL vs non-PRL's dysconnectivity

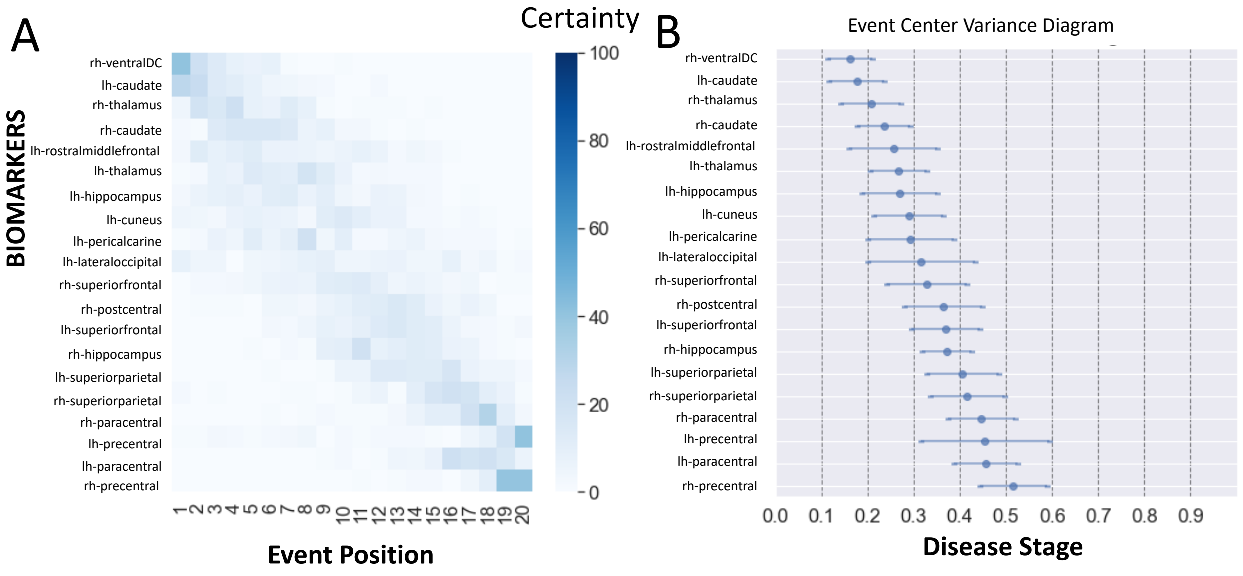

Supplementary Figure 3. (A) Positional variance diagram for cognitive impairment progression in pwMS for the 20 regions in which the disconnectivity metrics due to non-PRL were the most significantly different between CP and CI, where the CI group is identified as being impaired in at least 1 out of 3 cognitive tests. The maximum-likelihood sequence of abnormality is shown on the y-axis (top to bottom). Color intensity in each row indicates positional variance: the darker the color, the higher the confidence of the event position across 100 bootstraps. (B) The event-center variance diagram showing the standard error of estimated abnormality centers. rh/R= right hemisphere and lh/L=left hemisphere.

References

1. Glasser MF, Sotiropoulos SN, Wilson JA, et al. The minimal preprocessing pipelines for the Human Connectome Project. *Neuroimage*. 2013;80:105-124. doi:10.1016/j.neuroimage.2013.04.127
2. Jeurissen B, Tournier JD, Dhollander T, Connelly A, Sijbers J. Multi-tissue constrained spherical deconvolution for improved analysis of multi-shell diffusion MRI data. *Neuroimage*. 2014;103:411-426. doi:10.1016/j.neuroimage.2014.07.061
3. Tournier JD, , F. Calamante and a. C. Improved probabilistic streamlines tractography by 2 nd order integration over fibre orientation distributions. *Ismrm*. 2010;88(2003):2010.
4. Smith RE, Tournier JD, Calamante F, Connelly A. SIFT2: Enabling dense quantitative assessment of brain white matter connectivity using streamlines tractography. *Neuroimage*. 2015;119:338-351. doi:10.1016/j.neuroimage.2015.06.092
